# Ear-EEG sensitivity modeling for neural sources and ocular artifacts

**DOI:** 10.3389/fnins.2022.997377

**Published:** 2023-01-09

**Authors:** Metin C. Yarici, Mike Thornton, Danilo P. Mandic

**Affiliations:** Communications and Signal Processing, Electronic and Electrical Engineering, Imperial College, London, United Kingdom

**Keywords:** ear-EEG, forward modeling, blinks, vertical saccades, horizontal saccades, EEG artifacts, neural sources

## Abstract

The ear-EEG has emerged as a promising candidate for real-world wearable brain monitoring. While experimental studies have validated several applications of ear-EEG, the source-sensor relationship for neural sources from across the brain surface has not yet been established. In addition, modeling of the ear-EEG sensitivity to sources of artifacts is still missing. Through volume conductor modeling, the sensitivity of various configurations of ear-EEG is established for a range of neural sources, in addition to ocular artifact sources for the blink, vertical saccade, and horizontal saccade eye movements. Results conclusively support the introduction of ear-EEG into conventional EEG paradigms for monitoring neural activity that originates from within the temporal lobes, while also revealing the extent to which ear-EEG can be used for sources further away from these regions. The use of ear-EEG in scenarios prone to ocular artifacts is also supported, through the demonstration of proportional scaling of artifacts and neural signals in various configurations of ear-EEG. The results from this study can be used to support both existing and prospective experimental ear-EEG studies and applications in the context of sensitivity to both neural sources and ocular artifacts.

## Introduction

Electroencephalography (EEG) is a brain monitoring method that utilizes non-invasive electrodes placed on the scalp surface to extract electrical neural activity. The most common use-cases of EEG are clinical; these involve the localization and characterization of seizures related to epilepsy (Noachtar and Rémi, 2009) and the objective assessment of hearing ability in infants (Schulman-Galambos and Galambos, 1979). However, since modern EEG hardware is available in a miniaturized, portable form Abiri et al. (2019) and Wolpaw et al. (2002), EEG has also attracted attention in multiple real world applications of brain monitoring, such as brain computer interfaces (BCI).

Conventionally, EEG is recorded through an array (montage) of electrodes placed across the entire scalp, termed a scalp EEG montage. As a result of the wide coverage of the human scalp achievable with a scalp EEG montage, conventional EEG offers good spatial sensitivity to a variety of neuronal activity from across the brain surface. However, conventional scalp EEG is not suited for wearable applications as a result of the difficulty of integration of the hardware with everyday activity. Specifically, conventional scalp EEG is cumbersome, obtrusive, time-consuming to set up, difficult to use without specialist supervision, and introduces unwanted stigma for patient-populations (Casson, 2019). For these reasons, alternative, miniaturized, and wearable EEG montages which address these shortcomings hold much promise.

**Figure 1:**
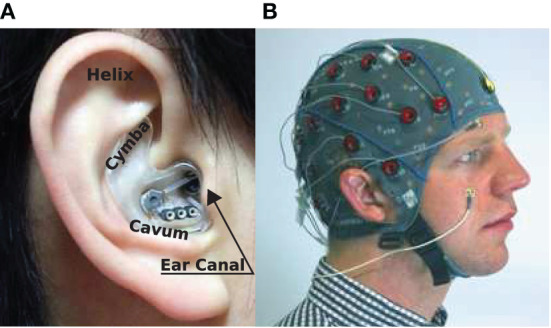
EEG sensing technologies: **(A)** In-ear-EEG device mounted on the right ear of a subject. Common locations for electrodes on the ear canal, concha (cavum and cymba), and helix are shown (Looney et al., 2012). **(B)** Standard scalp EEG cap and electrodes mounted on the head of a subject.

One such candidate is ear-EEG (Looney et al., 2011), which employs a small number of electrodes which measure EEG from the surface of the skin on the outer-ear (Mikkelsen et al., 2015). Importantly, ear worn devices are familiar, naturally discreet, unobtrusive, non-stigmatizing, and potentially easy-to-use, thus providing a convenient base for wearable health monitoring platforms (see Figure 1). Ear-EEG has been shown to be a reliable alternative to scalp EEG in several settings; sleep stage classification (Mikkelsen et al., 2017; Nakamura et al., 2017b), drowsiness onset detection (Nakamura et al., 2018), objective hearing threshold estimation (Bech Christensen et al., 2018), bio-metric authentication (Nakamura et al., 2017a), epileptic waveform detection (Zibrandtsen et al., 2017), brain-computer-interfaces (Goverdovsky et al., 2017; Yarici et al., 2021), and emotion recognition (Athavipach et al., 2019). Additionally, the susceptibility of ear-EEG to various artifacts has also been characterized experimentally for auditory neural activity detection in the presence of head, eye, and jaw movements (Kappel et al., 2017).

The source-sensor relationship is an important concept for EEG technologies. The source-sensor relationship is a characterization of the sensitivity of a sensor, or an EEG channel, to the signal sources of interest, which are typically neural current dipoles (Grech et al., 2008). In practical terms, knowledge of the source-sensor relationship not only facilitates the optimization of EEG channel configuration for a particular neural source, but can also provide rigorous, theoretical evidence for inferences drawn from weak experimental data, for example data that is collected *via* a low number of sensors and in noise-prone scenarios (Rush and Driscoll, 1968; Coburn and Moreno, 1988; Mosher et al., 1992). For wearable EEG technologies such as ear-EEG, signal-to-noise ratios (SNR) are often low in practice, which poses great challenges for the adoption of such wearable technologies in many scenarios. If the source-sensor relationship were known for wearable devices, the choice and design of the device could be optimized for particular applications.

Physics modeling of the propagation of neural potential to EEG sensors, commonly termed forward modeling, can be used to estimate the source-sensor relationship for various configurations of EEG (both montages and channels). In this paradigm, the electric potential on the surface of the scalp arising due to a current dipole source within the brain volume is estimated by applying Maxwell's equations to a structurally accurate dielectric model of the head (Sarvas, 1987), termed a volume conductor model. High resolution imaging, such as magnetic resonance imaging (MRI) scans or computerized tomography (CT) scans enable the construction of detailed volume conductor models. Unlike experimentation, forward modeling allows for the testing of a larger number of channels and sources in a time-efficient way.

Some limited forward modeling work related to ear-EEG has been reported in the literature. In an ear-EEG modeling study Kappel et al. (2019), demonstrated the feasibility of source localization using in-ear-EEG and provided highly detailed subject specific forward models that were created by scanning each subject's head anatomy. However, the source-sensor relationship for in-ear-EEG was not evaluated in detail. In a more detailed analysis, Meiser et al. employed forward modeling in order to compare the sensitivity of scalp EEG with cEEGrid–an alternative ear-EEG method which utilizes the skin surface surrounding the ear (Meiser et al., 2020). The study presented in this paper aimed to establish a detailed source-sensor relationship for various configurations of ear-EEG.

While the sensitivity to neural sources is a key determining factor in the reliability of an EEG technology, equally important is its robustness in the presence of artifacts. Despite the fact that the physics mechanisms underlying most common EEG artifacts have been long established [motion (Oliveira et al., 2016; Symeonidou et al., 2018), eye movement (Gratton, 1998; Joyce et al., 2004; Roy et al., 2014), muscle activation (Ma et al., 2012; Muthukumaraswamy, 2013; Richer et al., 2019), and electrical interference (Webster, 2009)], to date, there is no theoretical study of such artifacts in ear-EEG. In this paper, as well as exploring the source-sensor relationship for various neural sources, we aim to provide a detailed characterization of the source-sensor relationship for various ocular sources of artifacts, utilizing equivalent current dipole data for blinks, vertical saccades, and horizontal saccades [collected by Lins et al. (1993a)]. This paper aims to highlight the utility of approaching ear-EEG equipped with theoretical knowledge of not only neural source sensitivity, but also that for sources of artifact.

## Methods

Modeling was conducted in COMSOL Multiphyiscs^®^–a multi-physics modeling platform which enables finite element electromagnetic modeling in multiple physics domains (COMSOL, 2022). Geometric and dielectric human tissue data from the Information Technologies in Society Foundation (IT'IS; Gabriel, 1996; Iacono et al., 2015) was used to build an accurate (dielectric) model of the human head and ears to be used within COMSOL. The following section describes the structure of the model and the implementation of physics modeling within the COMSOL software.

### Forward modeling

For EEG modeling, the standard volume conductor–forward modeling approach was employed (Sarvas, 1987). The same approach was also employed for ocular artifact modeling. The utilization of such an approach for ocular source modeling is motivated by the fact that the ocular sources used within this study were described as equivalent current dipoles, which were discerned through standard EEG inverse modeling procedures in Lins et al. (1993a).

In this work, potential on the surface of the skin was extracted at several EEG locations of interest. For ear-EEG, four sites on each ear were sampled; the ear canal (*XEC*), concha cavum (*XCAV*), concha cymba (*XCYM*), and helix (*XHEL*), where *X* is either *L* (Left) or *R* (Right), denoting the specific ear. The placing of the electrodes is shown in **Figure 3A**. For scalp-EEG, a 64-channel montage was placed on the surface of the skin according to the 10–20 BESA (2022) convention. This configuration represents a commonly used montage within EEG research and provides a reasonable density of sampling and spatial extent of coverage on the head surface.

#### Volume conductor model

**Figure 2:**
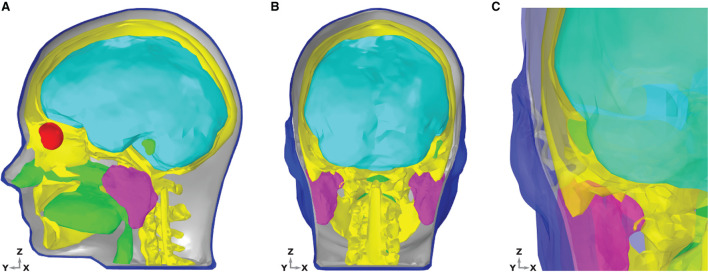
Dielectric distribution within the head and ear model. Different tissue groups are discernible by color; brain-cyan, bone-yellow, muscle-gray, air-green, vitreous humor-red, and skin-blue. **(A)** Sagittal section of the head showing the interior of the model. The skin and bones are hidden to the left of the mid line sagittal plane, such that the brain, eyes, glands, and internal air are clearly visible. **(B)** Coronal section of the head with the skin and bone hidden to the rear of the mid line coronal plane. **(C)** Coronal section of the head, zoomed in to the left ear region, viewed from a posterior position. The skin and bones are hidden to the rear of the mid line coronal plane. Tissues are transparent, to enable a detailed view of the entire ear and surrounding structures.

The aim of the present study is to provide the first ear-EEG source-sensor analysis for both neural and ocular sources. As such, in addition to standard EEG forward model structures (e.g., bone, brain, skin, and muscle) a volume conductor model which includes structures of the eyes and ears is necessary. Rather than provide subject specific modeling, the current study aims to provide generalizable modeling, such that the results could be applied to the general population. When building a generalizable model, anatomical geometry which closely resembles the average anatomy of the population is desirable, as this will maximize the portion of the population for which your predictions resemble experimental measurements. However, since geometries for the ear and eyes are not used in EEG forward models or detected in routine anatomical scans of the head, such average data for the eyes and ears has not been produced. In the place of data that is generalizable, the MIDA model has been used in the current study (Iacono et al., 2015). The MIDA model describes the head anatomy of a healthy adult male, is highly detailed, and includes 153 different tissues of the head (including the vitreous humor of the eyes and the skin surface of the ears). For the present model, an adapted and simplified geometry, including seven main tissues was created (see Figure 2). The tissues included were skin, including the inner and outer dermis of the whole head, neck, and outer ear; bone, including the skull and the C1–C3 vertebrae; brain tissues including gray matter, white matter, cerebellum, and brainstem surface; internal air (respiratory tracts and mastoid air-cells within the skull); parotid glands; vitreous humor (left and right eyes); and muscle. The muscle tissue was not designated a specific geometry, rather, surrounding regions of the other tissues were endowed with muscle tissue properties.

The tissue properties used within this model are provided in Table 1 and are drawn from the IT'IS tissue frequency database (Hasgall et al., 2022). Dielectric values at an excitation frequency of 10 Hz were used for this study in order to reflect a typical EEG frequency of interest.

**Table 1:** Dielectric properties of the tissue in the model taken from the IT'IS tissue frequency database (Hasgall et al., 2022).

	**Tissue**						
	**Bone**	**Skin (dry)**	**Brain (gray matter)**	**Muscle**	**Parotid glands**	**Eyes (vitreous humor)**	**Air**
σ(*S*/*m*)	2 × 10^−2^	2 × 10^−2^	2.9 × 10^−2^	2.2 × 10^−1^	6.7 × 10^−1^	1.5	0
ϵ_*r*_	5.5 × 10^−4^	1.1 × 10^3^	4 × 10^7^	2.6 × 10^7^	9.4 × 10^1^	9.9 × 10^1^	1

Values for conductivity (σ) and relative permittivity (ϵ_*r*_) at an excitation frequency of 10 Hz were used in the model.

The COMSOL Multiphysics software requires that geometry data does not exhibit non-manifold edges and self-intersecting faces. Therefore, the MIDA data was adapted and simplified during a pre-processing procedure in order to meet these compatibility requirements. Details of the pre-processing procedure are provided in the Supplemental material, while the entirety of the final model geometry will be provided upon request.

Within the COMSOL software, forward modeling was conducted within the electric currents interface (AC/DC Module) through the following methods. An equation for current density was used to solve for electric potential throughout the model:


(1) J=σE+jωD+Je,

where **J** is the total current density, σ is material conductivity, *E* is the electric field, *j* is the imaginary number, ω is the frequency of current, **D** is the displacement field, and *J_e_* is the external (source) current density (COMSOL, 2022).

The following equation of continuity was imposed across tissue boundaries:


(2) $$n_{2}(J_{1}-J_{2})=0,$$

where *n* is a unit vector that is normal to (and directed away from) the boundary and **J** is the electric current density. The indices 1, 2 describe the regions of space either side of the boundary. The head model was placed at the center of a large (*r* = 10 m) sphere with the dielectric properties of air (conductivity σ = 0 µS/cm, relative permittivity ϵ = 1). The surface of the sphere acted as the ground in the AC model. See Martinek et al. (2008), Pelot et al. (2018), and Seibt et al. (2019) for examples of volume conductor modeling within COMSOL.

### Neural sources

**Figure 3:**
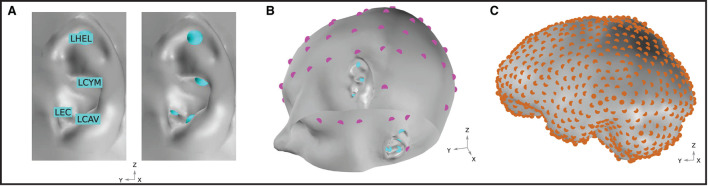
**(A)** Ear-EEG electrode locations and naming convention. Electrodes are shown for the left ear. An equivalent array is positioned on the right ear. **(B)** EEG electrode locations on the skin surface of the model. Magenta and cyan markers, respectively, indicate the location of electrodes in a 64-electrode scalp EEG montage and a left and right ear-EEG montage. Sixty-four scalp EEG electrodes are placed according to the 10–20 BESA (2022) convention. **(C)** Neural source space on the course-grained surface of the brain. Source locations are homogeneously distributed with a density of 1.5 cm^-2^ and are highlighted in orange.

The goal of the presented modeling was to evaluate the ear-EEG source-sensor relationship for a variety of realistic neural sources. The source space was restricted to the surface of the brain and comprised 990 homogeneously distributed source locations (Figure 3C). In this way, an exploration of the source-sensor relationship for a range of realistically located neural sources was achieved. Each location was occupied by a single source, enabling a fine-grained mapping of the brain surface. Each source was orientated at perpendicularly to the surface of the brain. Sources were modeled as point current dipoles; the location, orientation, and magnitude of which were specified (Sarvas, 1987).

#### Sensitivity maps

The source sensor relationship between a given EEG montage and the neural current sources is analyzed through the following method: for each of the 990 neural sources, the potential difference between all possible pairings of two electrodes (i.e., all possible bipolar channels) was determined. Of all these potential differences, the greatest unsigned potential difference is defined as the sensitivity of the EEG montage to the neural source in question. This method effectively identifies the best-case signal magnitude that can be measured by the EEG montage in question, for all neural current sources modeled. The neural sources were modeled as point current dipoles oscillating at 10 Hz. The entirety of the ear skin-surface is available to ear-EEG devices, meaning that EEG can be detected from anywhere on this surface through the use of multiple electrodes mounted on a single device (see the ear-EEG electrode locations in Figures 3A, B). The sensitivity maps in **Figure 5** display the sensitivities for each of the 990 modeled brain surface sources. The analysis was conducted for both unilateral and bilateral ear-EEG and scalp-EEG (64 channel 10–20 montage). Sensitivity data were transferred to visual representations of the sensitivity over the surface of the brain, or sensitivity maps, in MatLab^®^, using the source location and brain mesh data in the function trisurf. In order to enable clear visualization of the exponentially dynamic sensitivity map, sensitivities are plot in decibels (dB) relative to an arbitrary value, calculated through the equation 10*log*_10_(*V*/*V*_*reference*_), where *V* is the sensitivity value of interest and *V*_*reference*_ is the arbitrarily set reference value. In the chosen logarithmic scale, 10-fold differences in sensitivity are equal to ± 10 dB.

In addition to the sensitivity maps described above, relative sensitivity maps were created. In this analysis, the ear-EEG montage sensitivities were divided by the scalp EEG montage sensitivities, in order to enable calculation of the change in signal amplitude associated with the use of a particular ear-EEG montage over the 64-channel scalp-EEG montage. Once again, for each source, the scalp EEG and ear-EEG sensitivities are extracted from the optimal differential pair of electrodes within their respective montages. In this way, the expected increase/decrease in signal amplitude (signal gain/loss) associated with the use of both unilateral and bilateral ear-EEG over scalp EEG could be fairly examined. Meiser et al. introduced this method for the analysis of the cEEGrid source-sensor relationship in Meiser et al. (2020). The presently reported values are transformed into a logarithmic scale *via* the equation 10*log*_10_(*V*_*ear*_/*V*_*scalp*_), where *V*_*ear*_ and *V*_*scalp*_, respectively, are the optimal sensitivity values for the ear and scalp montages.

### Ocular artifact modeling

**Figure 4:**
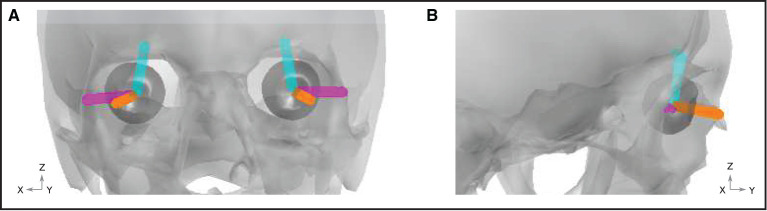
Ocular artifact dipoles. Orientation of the blink (orange), vertical (cyan), and horizontal (magenta) dipoles are shown within the geometry of the skull (gray) and eyes (dark gray). **(A)** Front view. **(B)** View from the right hand side of the head.

Artifacts arising due to blinks and eye movements can be explained in terms of the corneo-retinal dipole (CRD) field. This dipole field arises due to natural charge separation between the cornea and the retina. During eye movements, the CRD rotates around the center of the eyeball, resulting in a dipole current. During eye blinks, the conductive surface of the inner eyelid sweeps over the cornea, leading to current discharge, which can also be modeled as a current dipole. In Lins et al. (1993b), the authors performed dipole fits to electrooculographic data. They found that two-dipole fits (one dipole per eye) explained the data very well (explaining up to around 98% of the total variance). For each type of ocular artifact (vertical saccades, horizontal saccades, and blinks), the fitted dipoles shared approximately the same locations. For blinks, the dipoles were approximately aligned in the anterior-posterior direction. For vertical (horizontal) saccades, the dipoles were approximately aligned in the superior-inferior (lateral) direction. We therefore modeled each type of artifact using point current dipoles aligned with the directions reported by Lins et al. (1993a). Figure 4 displays a graphical representation of the dipoles, while numerical representations of the dipole vectors are provided in the Supplemental material.

For modeling ocular artifacts, a current dipole of fixed amplitude was used to model all three artifact types (blinks, vertical saccades, and horizontal saccades). The dipole amplitude was calibrated so that the resulting scalp topographies reflected the typical waveform amplitudes measured experimentally [for example, see Lins et al. (1993a)]. However, the values reported in this study are normalized and are therefore independent of the chosen dipole amplitude (the same results could be achieved with any dipole amplitude).

In our simulations, we neglected the rider artifact, which is a transient onset effect which occurs at the start of a vertical or horizontal saccade. Similar to blink artifacts, the rider artifact occurs because the eyelid lags behind the motion of the artifact, discharging slightly. In fact, the two blink dipoles can be used to explain the rider artifact.

For the purpose of validation of the ocular artifact modeling, simulations of scalp EEG sensitivities provided by the presented model were compared to measured data in Lins et al. (1993b); both sets of data are provided in Supplementary Table 3. The sensitivity of a number of scalp EEG channels ranging from frontal, central, occipital, and temporal sites were compared, where the sensitivities were expressed as percentages relative to values from reference channels; VEOG (for the blink and vertical saccade artifacts) and HEOG (for the horizontal saccade artifacts). Correspondence between the measurements in Lins et al. (1993b) and the presented simulations was calculated in terms of the mean error between sensitivity values. Good agreement was found between the measured and simulated potentials for all three of the investigated ocular artifacts, with a mean error of 3 % across all three artifacts.

## Results

### Ear-EEG sensitivity to neural sources

#### Sensitivity maps

**Figure 5:**
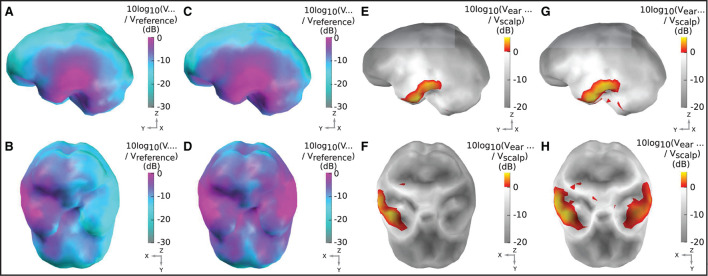
Sensitivity maps for ear-EEG: **(A, B)** Sensitivity map for a left ear unilateral ear-EEG montage. **(C, D)** Sensitivity map for a bilateral ear-EEG montage. **(E, F)** Relative sensitivity map for a left ear unilateral montage and a 64-channel scalp EEG montage. **(G, H)** Relative sensitivity map for a bilateral ear-EEG montage and a 64-channel scalp EEG montage. **(A–D)** High and low sensitivities, respectively, are represented by magenta and cyan shading. **(E–H)** Severe and moderate signal losses are displayed in gray and white, respectively. Signal gains are displayed in red and yellow. **(A, C, E, G)** Left brain surface. **(B, D, F, H)** Inferior surface of the brain.

Figures 5A, B displays the sensitivity map for a left ear unilateral ear-EEG montage (displaying the characteristic sensitivity profile for a single earpiece). As previously described, the sensitivities displayed for each individual dipole are extracted from the optimal differential pair of electrodes within the montage (for that dipole). This analysis enables examination of the full capability of a montage which is achievable through the optimal electrode pairing. The highest sensitivities of the unilateral montage were exclusively observed in the ipsi-lateral inferior and middle temporal lobe. Decreases in sensitivity were observed for regions surrounding the ipsi-lateral temporal lobe, with the lowest sensitivities observed for sources furthest away from the ipsi-lateral ear; in frontal, central, and posterior, and contra-lateral locations. For the bilateral montage, high sensitivities are observed across large portions of the left and right temporal lobe and even some surrounding regions, while the lowest sensitivities were observed for frontal, central, and posterior regions close to the mid-line sagittal plane.

The relative sensitivity of the unilateral montage is displayed in Figures 5E, F. As with the sensitivity maps described above, relative sensitivity values are calculated using the optimal electrode pairing within each montage. On the inferior ipsi-lateral temporal lobe, for a small collection of sources (2% of the total) there is moderate signal gain, with a median value of 2 dB (25/75th percentile: 1/4 dB). For the majority of the remaining sources, there is a severe signal loss. The median relative sensitivity for all sources for the unilateral ear-EEG montage is −17 dB (25/75th percentile: −20/−4 dB). For the bilateral montage, the regions of severe signal loss are reduced relative to the unilateral montage. The median relative sensitivity was found to be −10 dB (25/75th percentile: −15/−4 dB). A small portion (5%) of sources on both temporal lobes were detected with a signal gain, with the median of 2 dB (25/75th percentile: 1/3 dB).

#### Channel sensitivity analysis

**Figure 6:**
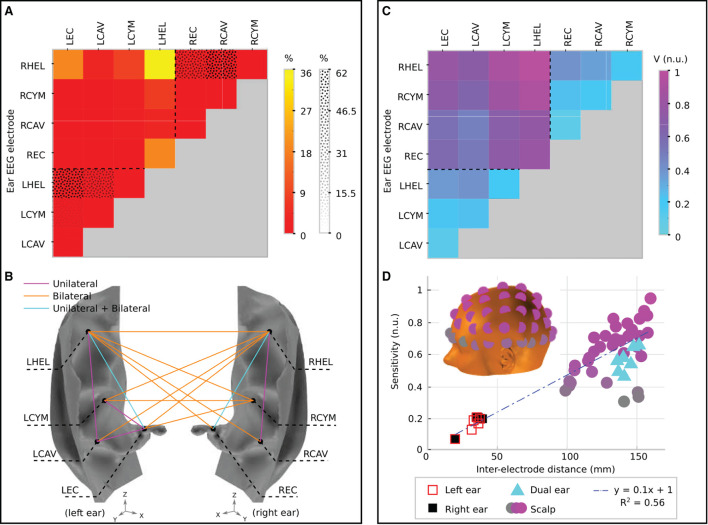
Channel prevalence and average sensitivity analysis for unilateral and bilateral montages of ear-EEG. **(A)** Channel prevalence for unilateral and bilateral montages of ear-EEG. Bilateral prevalence is shown in a color scale, while unilateral prevalence for the left and right ears is shown a in texture scale. Dashed lines separate single ear and bi-ear values on the heat-map. **(B)** Channel prevalence for unilateral and bilateral ear-EEG montages visualized on the surface of the ear. For channels that recorded the highest sensitivity within a montage for at least one source (>0%), a colored line connects the channel's electrodes. Magenta lines connect channels that were prevalent within the unilateral (left or right) montage analysis; orange lines connect prevalent channels in the bilateral montage analysis; and cyan lines connect channels which appeared prevalent in both unilateral and bilateral montage analyzes. **(C)** Normalized average sensitivity for unilateral and bilateral montages. Dashed lines separate single ear and bi-ear values on the heat-map. Unilateral and bilateral ear-EEG signals were normalized with respect to the same value. **(D)** Relationship between normalized sensitivity and inter-electrode distance for both ear-EEG (unilateral and bilateral) and scalp EEG. Both scalp EEG and ear-EEG were normalized with respect to the same value. A linear fit of the data is shown in a blue dashed line. Scalp EEG channel locations are indicated in the inset and colored corresponding to their sensitivity values. All of the channels that are displayed are referenced to the average of the sensitivities of the mastoid electrodes. The location of the left mastoid electrode is indicated by a black circle on the inset, while the right mastoid electrode is hidden from view (on the equivalent position on the right side of the head).

In Figure 6, the sensitivities of ear-EEG channels are assessed individually. Channels were created between all possible pairings of electrodes within each ear-EEG montage (unilateral and bilateral). First, the prevalence of the channels is evaluated, where the prevalence is equal to the percentage of brain sources for which the channel in question recorded the highest sensitivity. The prevalence can be viewed to indicate the relative utility of a channel within its montage. This approach was introduced in Meiser et al. (2020), for channels within a unilateral cEEGrid montage. In the prevalence analysis, only sources for which at least one channel from within the montage recorded a sensitivity above a certain threshold were included in the analysis. For all montages, the threshold was set to 10% of the highest sensitivity from the scalp montage, therefore sources for which large reductions in amplitude were observed did not feature in the analysis [see Meiser et al. (2020) for a similar methodology]. The use of thresholding was motivated by the fact that for sources which are poorly detected, knowledge of the channel that recorded the highest sensitivity is not informative.

Figure 6A displays the channel prevalence in the form of a heat map for both unilateral and bilateral montages (indicated by a dotted pattern and color shading, respectively). Within the left unilateral montage (bottom left of the heat map, separated by a dashed line) the helix to ear canal channel recorded the maximum sensitivity for 56% of selected sources. Helix to cavum, and cymba to ear canal were the next most prevalent (30 and 10%, respectively), followed by cavum to ear canal (3%). Helix to cymba and cymba to cavum failed to record the highest sensitivity for a source. Within the right ear unilateral montage (top right of the heat map, separated by a dashed line) the helix to cavum was the most prevalent (62%), with the helix to ear canal recording the highest sensitivity for the remaining sources (38%). Within the bilateral montage, which included all available electrodes on both the left and right ears, the left helix to right helix channel recorded the most maximum sensitivities (36%) for a single channel, while the bi-ear helix to ear canal channels (left helix - right ear canal/right helix to left ear canal) recorded the maximum sensitivity for 20% of sources each (Figure 6A). The left helix to right cymba and right helix to left cymba were the next most prevalent, recording 10 and 9% of maximum sensitivities, respectively. Figure 6B displays the complete set of channels which recorded the maximum sensitivity at least once. Dominance of the bilateral channels is clearly observed.

To supplement the channel prevalence analysis, the average sensitivity for each ear-EEG channel was also calculated (Figure 6C). As with the channel prevalence analysis, average sensitivities were based only on sources which satisfied the threshold condition described above. Values of average sensitivity are provided in normalized units (n.u.)–whereby unilateral and bilateral ear-EEG were normalized with respect to the same value: the maximum average ear-EEG sensitivity from within the unilateral and bilateral montages. For the average sensitivities, a linear scale was sufficient to reveal meaningful trends, i.e., the values reported are calculated *via* the equation *V*_*ave*_/*V*_*max*_, where *V*_*ave*_ is the average sensitivity of the channel in question, and *V*_*max*_ is the maximum average sensitivity. The helix to helix channel recorded the highest average signal amplitude (1 n.u.), however the majority of bi-ear channels recorded similar average amplitudes (>0.6 n.u.). The single ear channels exhibited lower average signal amplitude (<0.3 n.u.); the lowest average amplitude was recorded by the left and right single ear cavum to ear canal channels (<0.1 n.u.).

In order to further characterize ear-EEG, for a selection of ear- and scalp EEG channels, channel sensitivities were plotted against respective inter-electrode distance (Figure 6D). In order to comparison between scalp EEG and ear-EEG, the sensitivity of each channel was measured as the number of sources for which the channel sensitivity exceeded the previously described threshold. Ear and scalp channels were normalized with respect to the same value - the maximum sensitivity from within the ear-EEG and scalp EEG montages. For the scalp EEG channels, a linked mastoid referencing system was used, while for ear-EEG, left and right ear channels were referenced to the ipsi-lateral helix, and bi-ear channels to the contra-lateral helix. The inter-electrode distance for the linked mastoid referenced scalp EEG channels was calculated as the average of the distance of the primary electrode from both mastoid electrodes.

Both the sensitivity and inter-electrode distance of single ear channels are lower than those of the bi-ear and scalp EEG channels, leading to the ratio of mean channel sensitivity for left ear, right ear, and bi-ear-EEG relative to the mean channel sensitivity of scalp EEG, respectively, of 0.3, 0.3, and 0.9. A linear trend with equation of best fit, *y* = 0.1*x*+1, (*R*^2^ = 0.56) was found for the data displayed in the plot, where *y*= sensitivity, *x*= inter-electrode distance, and *R*^2^ is the goodness-of-fit measure for the linear trend.

### Ocular artifact modeling

**Figure 7:**
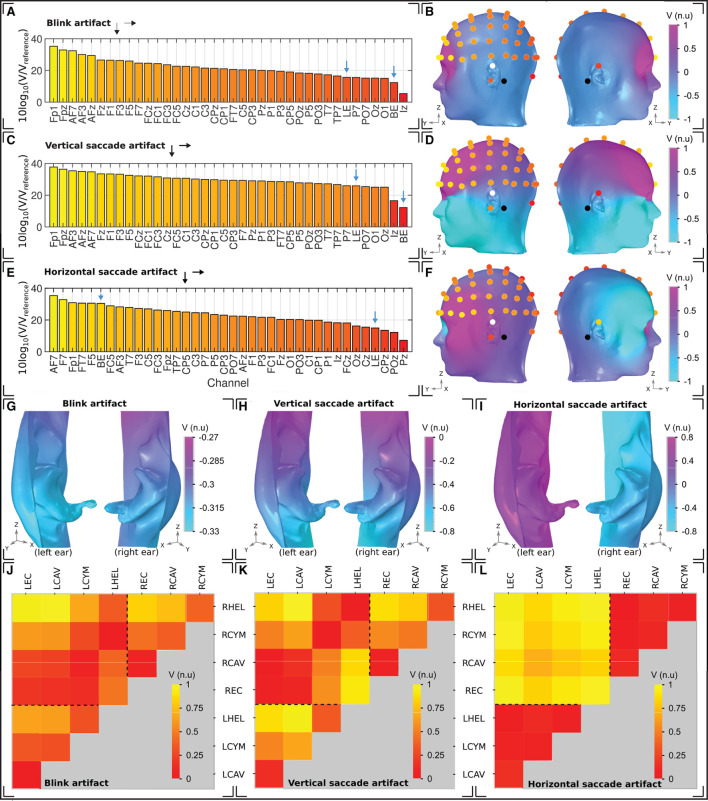
Ocular artifacts. **(A, C, E)** Sensitivities of the left hemisphere scalp EEG channels in addition to a unilateral left ear (LE) and bilateral (BE) ear-EEG channel, for **(A)** blinks, **(C)** vertical saccades, and **(E)** horizontal saccades. **(B, D, F)** Head surface potential topography and EEG sensitivities in normalized units (n.u.) arising due to **(B)** blinks, **(D)** vertical saccades, and **(F)** horizontal saccades. Scales are normalized uniformly across each of the head surface topographies such that an inter-artifact comparison is possible. White (black) circles indicate ear (scalp) reference electrodes. **(G–I)** Left and right ear surface potential topography arising due to **(G)** blinks, **(H)** vertical saccades, and **(I)** horizontal saccades. Potential scales are shared with the scalp topographies, however the color scale has been changed to enable clear visualization of the potential topography over each ear surface. **(J–L)** Sensitivity in normalized units (n.u.) of ear-EEG channels arising due to **(J)** blinks, **(K)** vertical saccades, and **(L)** horizontal saccades. The potential scale has been normalized for each individual plot; potential scales are not shared between artifacts.

The sensitivity of different configurations of ear-EEG in the presence of three common types of ocular artifact, blinking, vertical saccade, and horizontal saccade, was investigated. The sensitivity of ear-EEG to ocular artifacts was bench-marked against that of scalp EEG in Figures 7A, C, E. Specifically, sensitivities for two ear-EEG channels; single-ear left helix to left ear canal (LEH-LEC) and bi-ear left helix to right helix (LHEL-RHEL) are displayed alongside sensitivities of multiple scalp EEG channels that were referenced to the linked mastoids. Since the scalp EEG channel sensitivities are approximately symmetric about the midline sagittal plane, the inclusion of only a single hemisphere's (left) EEG channels was sufficient to capture the general variations in scalp EEG sensitivities to ocular artifacts. As a result of the large range (multiple orders of magnitude) of channel sensitivities, values in dB were calculated *via* the equation [10*log*_10_(*V*/*V*_*reference*_)], where *V* is the channel sensitivity of interest, and *V*_*reference*_ is equal to an arbitrarily set reference value. For all three artifacts, sensitivity values are reported with respect to the same reference value to enable between-artifact comparison.

For the blink artifact (Figure 7A), the maximum scalp EEG sensitivity was recorded by the FP1 channel and the lowest by the Iz channel (33 dB difference). Regarding ear-EEG, the blink artifact resulted in a larger potential difference in the LHEL-LEC channel relative to the LHEL-RHEL channel, with a difference between the sensitivities of 3 dB. Relative to scalp EEG, the LHEL-LEC channel was most similar (<1 dB difference) to scalp channels with a lateral positioning (P7, TP7), while the LHEL-RHEL channel was most similar (<1 dB difference) to the posterior scalp channel O1. The LHEL-LEC and LHEL-RHEL sensitivities were among the least sensitive out of the selection of scalp and ear-EEG channels analyzed (7th and 2nd least sensitive out of 37 channels, respectively). The resultant potential topography on the surface of the head with overlaid EEG channel topography is shown in Figure 7B, while a magnified view of the potential on the ears is provided in Figure 7G.

For the vertical saccade artifact, the maximum scalp EEG sensitivity was recorded by the FP1 channel and the lowest by the Iz channel; (26 dB difference). The LHEL-LEC channel was most similar to lateral and posterior scalp EEG channels, P7 and POz (<26 dB difference), while the LHEL-RHEL channel was most similar to the posterior inferior scalp EEG channel, Iz (<5 dB difference). The LHEL-LEC channel was 14 dB more sensitive relative to the LHEL-RHEL channel. The LHEL-LEC and LHEL-RHEL sensitivities were among the least sensitive out of the selection of scalp and ear-EEG channels analyzed (6th and 1st least sensitive, respectively). The topography of the vertical saccade potential over the surface of the scalp and ear, respectively, are shown in Figures 7D, H.

For the horizontal saccade artifact, the maximum scalp EEG sensitivity was recorded by the AF7 channel and the lowest by the central and parietal scalp EEG channels along the midline sagittal plane, Cz and CPz (<2 dB difference), while the LHEL-RHEL channel was most similar to frontal-lateral and central-lateral channels, F5 and FC5 (<2 dB difference). The LHEL-LEC channel was 15 dB less sensitive relative to the LHEL-RHEL channel. The LHEL-LEC sensitivity was among the least sensitive out of the selection of scalp and ear-EEG channels analyzed, while the LHEL-RHEL sensitivity was among the most sensitive (4th least sensitive and 6th most sensitive, respectively). The topography of the horizontal saccade potential over the surface of the scalp and ear, respectively, are shown in Figures 7F, I. The mean, maximum, and minimum sensitivity for the blink, vertical saccade, and horizontal saccade artifact were also calculated for each montage and are presented in Table 2. For each artifact, Figures 7J–L display the normalized sensitivities for the various ear-EEG channels, where for each artifact, the channel sensitivities were normalized *via* the equation *V*_*channel*_/*V*_*max*_, where *V*_*channel*_ is the sensitivity of the channel in question, and *V*_*max*_ is the maximum sensitivity from all evaluated channels.

**Table 2:** Sensitivity values for scalp EEG and ear-EEG montages in response to blink, vertical saccade, and horizontal saccade artifacts.

**Sensitivities to ocular artifacts:** 10***log***_**10**_(***V***/***V***_*****reference*****_) **(dB)**			
	**Ocular artifact**		
**EEG montage**	**Blink**	**Vertical saccade**	**Horizontal saccade**
Scalp (linked mastoid reference)	26 (35, 5)	31 (38, 17)	27 (35, 7)
Left and right unilateral ear-EEG	13 (16, 1)	24 (27, 15)	20 (22, 14)
Bilateral ear-EEG	13 (17, 2)	23 (26, 10)	30 (31, 29)

For each montage, mean values are reported alongside maximum and minimum values, respectively, in parentheses. Values are reported in dB, *via* the transform 10*log*_10_(*V*/*V*_*reference*_), where *V* is equal to the channel sensitivity, and *V*_*reference*_ is a reference value common to all artifacts and EEG montages/channels.

## Discussion

### Sensitivity to neural sources

The sensitivity of both unilateral (single ear) and bilateral (bi-ear) montages to neural sources across the entire brain surface was examined. While unilateral montages are confined to measuring potential differences over the small region of the ear, bilateral montages enable measurement between the left and right ears. In this way, the bilateral montage increases the inter-electrode distance, and therefore the potential difference, as a result of the physical laws governing EEG. Indeed, in Kappel et al. (2019), such differences are clearly observed between exemplar single channel lead fields for unilateral and bilateral ear-EEG. However, the present results for the montage sensitivities, which show the optimized sensitivity over a more diverse range of sources over the brain surface, reveal that several key, large scale variations in sensitivity for montages are similar in the unilateral and bilateral cases. For example, in (i) temporal lobe regions of highest sensitivity in close proximity to the ear electrodes and (ii) the regions of lowest sensitivity in proximity to the midline sagittal plane, the sensitivity profiles are similar. However, in between these regions, benefits of larger inter-electrode distance were clearly observed. Such differences between the unilateral and bilateral montages were observed for both the regular sensitivity maps (ear-EEG sensitivity) and the relative sensitivity maps (ear-EEG sensitivity bench marked against scalp EEG sensitivity).

Since EEG is conventionality recorded through scalp EEG, it is useful to compare the amplitude of the ear-EEG signal to that of scalp EEG. Therefore, relative sensitivity maps were also created. Ear-EEG produced an increase in signal amplitude in small regions in the temporal lobe, while adjacent regions mostly exhibited a moderate decrease in signal amplitude. In regions furthest away from the ear-EEG electrodes, the ear-EEG amplitude was shown to be considerably smaller than that of scalp EEG. The results for sources in and adjacent to the temporal lobe suggests that ear-EEG can be expected to record EEG amplitudes similar to those seen in scalp EEG in these regions. Since temporal lobe neural activity is known to correspond to important auditory and visuo-auditory processing, among other functionality, the use of ear-EEG in applications such as enhanced, “smart” hearing aids is strongly supported by these results. Indeed, reliable hearing threshold estimation on subjects with normal hearing and sensorineural hearing loss was demonstrated in two studies (Christensen et al., 2017; Bech Christensen et al., 2018), where comparable variance in scalp and ear-based estimations was found.

Sensitivity analysis of the various possible channels within a unilateral montage showed that the channels which maximize the space available on the ear surface, while also utilizing the helix position/electrode (at closer proximity to the brain), recorded the highest amplitude for the majority of sources. These channels were: the helix to ipsi-lateral ear canal and helix to ipsi-lateral cavum channels. These results are in line with experimental data in Kappel et al. (2016), where optimum reference configurations for the auditory steady-state response (ASSR) were investigated, and theoretical predictions in Meiser et al. (2020), where forward models of cEEGrid showed that channels maximizing the vertical distance between electrodes possessed the most favorable sensitivity profiles. For the bilateral montage, the same trend was observed, with the helix to contra-lateral ear canal channels and helix to contra-lateral helix channels producing the highest sensitivities. As such, in the scenario where a reduction of the ear-EEG montage size is desirable, the use of the helix and ear canal electrodes could be prioritized. However, despite the dominance of the helix and ear canal electrodes, in both the unilateral and bilateral montages, multiple electrode locations contributed to the highest sensitivity for at least one source, indicating that, while the area available on the ear surface is small, varied channel geometry within the small area is beneficial. In other words, several configurations of ear-EEG could be of use often in practice. Such benefits of varied channel geometry have been experimentally demonstrated in Kappel and Kidmose (2017), where high-density ear-EEG earpieces, with electrodes covering a large area of the ear surface, were tested in the presence of visual and auditory responses. The authors showed that for each different response, several locations across the ear could be used with comparable performance, while between responses, the optimal locations for EEG detection varied. Benefits of a high density ear-EEG array were also demonstrated in Kappel et al. (2019), where subject specific volume conductor model predictions and experimental high density ear-EEG data were shown to be in good agreement.

To provide further insight, the average sensitivity of the various possible channels within a bilateral and unilateral montage was also calculated. Calculations further supported the use of multi-electrode ear-EEG array, through comparable average sensitivities for geometrically similar channels. The expected signal amplitude of various ear-EEG and scalp EEG channels were then examined in relation to their inter-electrode distance. In general, for both scalp and ear-EEG, the expected signal amplitude was linearly proportional to inter-electrode distance. The revealed relationship between expected signal amplitude and inter-electrode distance could be used during the design process for various wearable/Hearable devices which utilize the ear and scalp surfaces. Further, it shows that even for ear-based EEG devices, amplitude will scale with area covered on the ear.

### Sensitivity to ocular artifacts

For prospective general purpose EEGs, the sensitivity to ocular artifacts is an important factor to consider. During most everyday activities, humans frequently blink and perform visual scans which involve horizontal and vertical saccades, with each instance of eye movement presenting a different artifact in the EEG signal. The sensitivity to ocular artifacts for ear-EEG and scalp EEG was demonstrated through both single channel sensitivity calculations and topographical plots of potential over the surface of the head. Ear-EEG sensitivities were calculated for a characteristic channel from both a unilateral montage (single ear) and a bilateral montage (bi-ear), while multiple linked mastoid reference scalp-EEG sensitivities were also calculated. Generally, the sensitivity to ocular artifacts for the single ear channel relative to the linked mastoid scalp EEG channels was observed to be low, evidenced through channel sensitivities of ear-EEG matching those of scalp EEG channels which are among the least severely affected by ocular artifacts. There was further evidence of low ear-EEG sensitivity in the topographical plots, where, relative to the scalp surface, the ear surfaces exhibited small ranges of potential. Experimentally measured EEG SNR deterioration caused by ocular artifacts was investigated in Kappel et al. (2017). For blink artifacts, deterioration in SNR was observed in multiple scalp electrodes, while for ear-EEG electrodes, no deterioration was detected. These results are in good agreement with the findings in this paper, as well as previous scalp EEG studies (Lins et al., 1993b; Gratton, 1998; Joyce et al., 2004; Roy et al., 2014). For vertical and horizontal eye movements, unexpected SNR deterioration patterns in scalp-EEG were observed. While vertical and horizontal saccades were performed by subjects at a rate of once every 4 s (0.25 Hz), the only deterioration in EEG for the scalp electrodes was observed in the theta- to gamma-EEG range (4–30 Hz). Since EMG activity is most prominent in higher frequency ranges, the authors attributed the unexpected patterns of EEG deterioration to inadvertent muscle contractions during the measurements, as opposed to the investigated ocular artifacts. Although deterioration in the delta- to gamma-EEG range (0–30 Hz) was observed for ear-EEG, further measurements which support these findings are required. The experimental difficulties highlighted in Kappel et al. (2017) demonstrate the importance of biophysics modeling approaches, which enable the investigation of electrophysiological sources in isolation.

For the bi-ear ear-EEG, the sensitivity was also low for the blink and vertical saccade - evidenced through similarity to low sensitivity scalp EEG channels and minimally varying topography over the ear skin-surfaces. However, an exception occurred during the horizontal saccade, when sensitivities equal to those of the worst affected frontal-lateral scalp EEG channels were observed for the bilateral channel. Such results are explained by the topography for the horizontal artifact (Figures 7F, I), which reveals a large potential difference between the two ear surfaces. Measurements of such bi-ear ear-EEG data that could be compared to the presented simulations have not yet been reported.

The variation in ocular artifact amplitude between the various possible ear-EEG channels was also examined. The map-plots of sensitivity show expected trends, where channels with larger inter-electrode distance have generally larger amplitudes. However, there are also variations in sensitivity which arise due to variations in channel orientation relative to the dipole field. For example, for the vertical saccade artifact, there is a 14 dB increase in sensitivity for the LHEL-LEC channel relative to the LHEL-RHEL channel, despite a much smaller inter-electrode distance (Figure 7C). Such characteristic variations within the ear-EEG montages could be used in the detection of artifacts, and highlight another benefit of utilizing a multi-electrode ear-EEG array, as opposed to single channels. Ear-EEG data that could be compared to the presented channel sensitivity analysis of ocular artifacts have not yet been reported.

### Suggestions for ear-EEG

For the first time, a systematic and detailed analysis of the ear-EEG source-sensor relationship was provided for a wide variety of neural sources from realistic locations, while various configurations of ear-EEG were considered. In addition, novel ear-EEG source-sensor relationships for vertical saccade, horizontal saccade and blink-related ocular artifacts have been established. With regard to both EEG detection and sensitivity to ocular artifacts, such source-sensor mapping, while serving to provide novel insight into the ear-EEG sensitivity profile, could also be used to support experimental measurements from ear-EEG in existing literature or in prospective studies. Additionally, the methods employed within this study can be adopted with reasonable ease by researchers for the purpose of conducting new ear-EEG simulations.

Simulations of neural source sensitivity have conclusively supported the use of both unilateral and bilateral ear-EEG montages for the detection of neural activity originating from within the temporal lobe. In both the unilateral and bilateral cases, the ear-EEG was estimated to record higher or similar amplitudes to conventional scalp EEG within these regions. This suggests that existing protocols for EEG detection could be used with ear-EEG, without the need for considerable changes to the protocol, and with equally likely success. In fact, as a result of the wearability of ear-EEG, existing auditory EEG protocols could feasibly be enhanced to include more novel real world recording scenarios, as demonstrated in a “smart helmet with ASSR” study (Von Rosenberg et al., 2016), where auditory brain responses were recorded from a subject while riding a bike. Since moderate decreases in amplitude were also observed for ear-EEG in brain regions adjacent to the temporal lobe, covering a variety of neural function, there is also support for similar use of ear-EEG in a wider range of applications.

Despite the models predictions of low amplitude ear-EEG for sources located in areas furthest away from the temporal lobes, e.g., the posterior regions of the brain, there is experimental support for ear-EEG detection of neural activity from such regions, such as the visual cortex in the occipital lobe (e.g., Kidmose et al., 2013; Goverdovsky et al., 2017). In these experimental studies, the successful detection of visual ERPs through ear-EEG have been possible despite smaller amplitudes (as predicted within the simulations within this paper). A likely reason for this is the lower noise amplitudes within the ear-EEG, where noise originated both from untargeted brain signals and other endogenous sources such as eye movements and muscle activity. This theory is backed by the simulated examples of decreases in both signal and noise amplitude in ear-EEG in this paper. Indeed, an approximately proportional scaling of EEG and artifact sensitivities is observed for both unilateral and bilateral montages with each ocular artifact (compare the analysis of inter-electrode distance and sensitivity in Figure 6D with the ocular artifact channel sensitivities and topographical plots in Figures 7A–F). Such results support the use of ear-EEG in the detection of neural activity from regions further away from the ear, despite lower signal amplitude.

### Limitations and future work

The first limitation of the presented study is the absence of absolute sensitivity predictions (predictions of the amplitude of ear-EEG recordings). However, the amplitude of EEG signals is likely to change as a result of many factors, such as the skin-electrode contact quality or the size of the group of neurons recruited for the response. In the absence of absolute sensitivity predictions, the approach adopted within this study focused on relative differences between various channels. In this way, meaningful comparisons were drawn without requiring knowledge of absolute sensitivity values. Relative amplitudes are also useful in practice, since, regardless of the absolute amplitude of the response, the alignment between model predictions and measured relative amplitude between two channels can serve as an indicator for reliable EEG recordings. Nevertheless, absolute predictions could be made possible through calibration of the model with experimental measurements.

For a generalizable EEG model, geometry resembling that of the population average is desirable, since the aim of the modeling is to provide results that can be applied to most subjects. However, average head geometry data including tissues such as the eyes and ears, which are required for neural and ocular ear-EEG sensitivity modeling, does not exist. Therefore, the present volume conductor model was built using single subject head geometry data from the widely accepted MIDA model (Iacono et al., 2015). In order to mitigate the use of subject specific geometry, the analysis conducted in this paper reflected that of other generalizable EEG models, which focus on large-scale, general variations in geometry that are expected to be shared between large portions of the population. Moreover, considering that (i) the current model was based on the anatomical geometry of a subject with no known physical abnormalities, and (ii) inter-subject anatomical variability is sufficient to substantially limit the generalizability of average anatomical geometry, the presently used single-subject geometry is likely to suffice in producing generalizable results, provided that the interpretation of the results are within the bounds of generalizable modeling.

A 64-channel scalp EEG configuration was considered in this study. The scalp electrodes were placed according to the standard 10–20 system. We compared the source-sensor relationship of this scalp montage to that of the ear-EEG montage. This particular arrangement of scalp EEG electrodes was selected because of its high adoption rate in research, its relatively high density, and its wide spatial distribution across the scalp. However, it does not include many electrodes around the ears. Therefore, the reader should note that other scalp montages which contain a higher density of electrodes around the ear regions would likely produce sensitivity profiles more similar to that of ear-EEG for temporal lobe regions, and could probably perform with less, or even no signal loss (relative to ear-EEG) in temporal regions (Figures 5E–H).

A systematic analysis of the sensitivity to source orientation, position, and distance, such as that presented in Meiser et al. (2020) for cEEGrid, has not been the aim of the present study, which does not consider the analysis of the associated variations in source characteristics. One way in which such an analysis could be achieved with the current model is, for each of the 990 dipoles simulated in this study, to test varying orientations. Since few assumptions about the exact cortical folding structure of the brain would be made when employing this method, the absence of subject specific anatomy would be partially mitigated, enabling the model to maintain a level of generalizability.

In principle, the presented volume conductor modeling framework could be used to investigate the sensitivity of wearable EEG montages to muscle artifacts, for example by placing current sources in the locations from which EMG signals originate. An example of accurate volume conductor modeling of muscles has been provided by Pereira Botelho et al. (2019). The reader should be aware that the modeling method employed within Pereira Botelho et al. (2019) utilizes both the muscle fiber geometry and motor unit activation patterns for the muscle of interest, in addition to a modified current-source modeling approach which exploits the reciprocity theorem (Plonsey, 1963; Rush and Driscoll, 1969). Such methods were employed to gain accurate predictions, and in an efficient manner (the reciprocity theorem applied to single channel predictions enables reduced computation times for a large number of sources). Therefore, while EMG simulation within the current framework is feasible, more detailed current modeling than that which is shown here might be required for meaningful results.

For artifacts that do not arise as a result of an internal source of electric field, for example motion and external field artifacts, the presented modeling framework would require modification, such that the relevant physics is incorporated into the model. An advantage of simulating through COMSOL is that an existing model can be adapted and used in simulations of various physics domains, for example mechanics or electric fields and circuits. As such, it is possible that the presented model could be modified to incorporate simulations of head and electrode mechanics during motion, or external field interference.

## Conclusion

Novel insights into the ear-EEG source sensor relationship for both neural and ocular sources have been provided, through comparisons of single channel and montage sensitivity profiles for ear and scalp EEG. The results have provided conclusive evidence for the use of ear-EEG in applications concerning the monitoring of neural activity originating from within the temporal lobes, for both unilateral and bilateral montages of ear EEG, while evidence has been provided for equal SNR between ear-EEG and scalp EEG in the presence of ocular artifacts. The reported results could also be used as a reference for various neural and ocular sources, supporting both existing and prospective experimental ear-EEG studies. Future work will look to exploit the utility of the presented physics modeling to provide further insight into the sensitivity of ear-EEG to both neural and a variety of artifact sources.

## Data availability statement

The raw data supporting the conclusions of this article will be made available by the authors, without undue reservation.

## Ethics statement

Written informed consent was obtained from the individual(s) for the publication of any potentially identifiable images or data included in this article.

## Author contributions

Neural source modeling was devised and analyzed by MY and conducted by MY and MT with supervision from DM. Ocular source modeling was devised, conducted, and analyzed by MY and MT with supervision from DM. The manuscript was prepared by MY and MT and edited by all authors. All authors contributed to the article and approved the submitted version.
