# Supplementary material for: Ear-EEG sensitivity modeling for neural sources and ocular artifacts
Source: Front Neurosci. 2023 Jan 9;16:997377. doi: 10.3389/fnins.2022.997377 (PMC9868963; doi:10.3389/fnins.2022.997377)
Supplement: Supplementary file 1 [file Data_Sheet_1.docx]

**Details of the pre-processing procedure for the volume conductor model**

Forward modelling within COMSOL is based on the application of physics equations to geometrical objects. As such, geometrical artifacts such as non-manifold edges and self-intersecting surfaces are incompatible with forward modelling within COMSOL. The high spatial resolution of the MIDA model includes vast amounts of the aforementioned geometrical artifacts, in addition to sub-millimetre surface detail which is not required for the purpose of EEG forward modelling. In order to remove both artifacts and unnecessary surface detail, data were imported into Autodesk Meshmixer and edited. Editing was conducted via a mixture of mesh operations including smoothing, remeshing, reduction, removal, filling, Boolean joining, and grouping. The final edited geometries are smooth, shell-like structures which describe the outermost surfaces of a tissue domain. In order to build each of the final geometries, several of the segmented tissues from the MIDA model were used (see Table S1). Once the editing was complete, the geometries were subsequently imported into COMSOL. Utilising a 'finer' mesh size in COMSOL, the final volume conductor model, including the enclosing sphere, consisted of a total of 80874 face elements, and 63046 vertex elements.

Table S1. Tissues in the model and corresponding MIDA data.

|  | Tissue | | | | | |
| --- | --- | --- | --- | --- | --- | --- |
|  | Bone | Skin | Brain | Parotid Gland | Eye (vitreous humour) | Air |
| MIDA files | Skull diploe,  Skull inner table,  Skull outer table,  Skull,  Teeth,  Spinal cord,  C1 – C3 | Adipose tissue,  Subcutaneous adipose tissue,  Auditory canal,  Auricular cartilage,  Epidermis and dermis | Gray matter,  White matter,  Cerebellum gray matter,  Cerebellum white matter,  Brainstem midbrain,  Brainstem pons | Parotid gland | Eye vitreous | Mastoid,  Nasal pharynx,  Oral cavity |

**Table of ocular dipole orientations**

Table S2. Ocular dipole orientations: Orientation of dipoles are given in unit vectors. The z-axis is in approximate alignment with the inferior-superior axis of the head.

|  | Ocular artifact | | | | | | | | |
| --- | --- | --- | --- | --- | --- | --- | --- | --- | --- |
|  | Blink | | | Vertical saccade | | | Horizontal saccade | | |
|  | x | y | z | x | y | z | x | y | z |
| Left eye | -0.304 | -0.937 | 0.183 | 0.183 | -0.159 | -0.97 | -0.988 | -0.156 | 0.017 |
| Right eye | 0.432 | -0.885 | 0.174 | -0.159 | -0.183 | -0.97 | -0.982 | 0.156 | 0.104 |

**Measured and simulated ocular artifact sensitivities in scalp EEG**

Table S3. Agreement between measured and simulated scalp EEG sensitivities. Measurements of scalp sensitivities in [1] (Table 4 – second experiment, for blinks and vertical saccades; Table 5 – second experiment, for horizontal sacacdes) are compared to the simulations of potentials from the COMSOL model presented in this paper. Measured data in [1] are provided as the mean (±standard deviation) of the percentage of potential in the scalp channels relative to reference channels; VEOG (for the blink and vertical saccade artifacts) and HEOG (for the horizontal saccade artifact). For the measured data in [1], a non-cephalic EEG reference was used; reference to the mean of an electrode placed on the right sternoclavicutar junction (at the top of the chest on the front surface of the body) and the nape of the neck. In COMSOL, only the nape of the neck was realistically modelled, therefore only this site was used as a reference. Since the potentials at both of the listed non-cephalic sites is extremely small, the difference between the reference configurations in [1] and in the present study is minimal. The mean error between the simulated and mean measured values is 3%.

|  | Blink (% of VEOG) | | Vertical saccade (% of VEOG) | | Horizontal saccade (% of HEOG) | |
| --- | --- | --- | --- | --- | --- | --- |
| Scalp EEG channel | Lins *et al*., 1993 | COMSOL | Lins *et al*., 1993 | COMSOL | Lins *et al*., 1993 | COMSOL |
| Fz | 18±4 | 19 | 18±4 | 25 | 0±5 | 4 |
| Cz | 8±4 | 7 | 10±4 | 16 | 0±5 | 4 |
| Oz | 4±3 | 2 | 5±4 | 7 | 0±3 | 2 |
| C3/4 | 8±3 | 6 | 10±4 | 16 | 4±5 | 7 |
| T3/4 | 5±2 | 5 | 6±3 | 11 | 8±5 | 9 |
| F7/8 | 17±4 | 10 | 15±5 | 14 | 22±7 | 31 |

**References**

[1] Lins, O.G., Picton, T.W., Berg, P. et al. Ocular artifacts in EEG and event-related potentials I: Scalp topography. Brain Topogr 6, 51–63 (1993). https://doi.org/10.1007/BF01234127
